# Proteomics analysis of body fluid exosomes of rheumatoid arthritis patients underwent oxhorn cupping therapy

**DOI:** 10.1371/journal.pone.0311526

**Published:** 2024-12-12

**Authors:** Du Leng, Chimedragchaa Chimedtseren, Tegexibaiyin Wang, Ruga Su, Saren Bao, Xiele Mo, Xigesaiyin Ge, Suna Cha, Runtulaguer Xi, Saqila Wu, RenGaoWa Sa, Jiaqi Zhao, Ren Na, Perenlei Molor-Erdene

**Affiliations:** 1 Xilinguole Meng Mongolian General Hospital, Xilinhaote, China; 2 Mongolian National University of Medical Sciences, Ulaanbaatar, Mongolia; 3 Affiliated Hospital of Inner Mongolia Minzu University, Tongliao, China; 4 Inner Mongolia Minzu University, Tongliao, China; Texas Tech University Health Science, Lubbock, UNITED STATES OF AMERICA

## Abstract

**Objective:**

The present study was undertaken to understand the multitarget mechanisms of oxhorn cupping therapy (OHCT) in treating rheumatoid arthritis by proteomic analysis.

**Methods:**

Thirty rheumatoid arthritis patients underwent OHCT and liquid (body fluid) accumulated in the cupping vessels was collected. Exosomes from the body fluid were isolated and characterized by transmission electron microscope (TEM). Particle size analysis, fluorescent labeling, and flow cytometry detection were also performed. Label-free quantitative proteomics analysis was used to detect differentially expressed proteins (DEPs). The Kyoto Encyclopedia of Genes and Genomes (KEGG) pathway enrichment, gene ontology (GO) enrichment, clusters of orthologous groups (COG), and protein-protein interaction (PPI) network were used to perform bioinformatics analysis of DEPs. Enzyme-linked immunosorbent assay (ELISA) was used to detect the key targets regulated by OHCT.

**Results:**

According to TEM images, the average size of exosomes in body fluid of RA patients underwent OHCT was 76.13 nm (5.27E+10 /mL). The positive rates of CD9, CD63, and CD8 were detected on the surface of body fluid exosomes. A total of 300 DEPs (58 up-regulated and 242 down-regulated) were identified between the pre-treatment and post-treatment stages. DEPs were related mostly to protein binding, focal adhesion, extracellular region, post-translational modification and signal transduction. KEGG pathway analysis showed a significant enrichment of DEPs in PI3K-Akt pathway and focal adhesion. Ten DEGs (ITGA5, ITGA4, ENG, MMP14, SERPINH1, THY1, TAGLN, ITGA1, IGF1, and ITGB5) were considered target genes according to PPI network analysis. ELISA showed a slight decrease in the serum levels of CDK1, ITGA5, ITGB5, and CD44 during and after treatment.

**Conclusions:**

Body fluid samples from RA patients treated with oxhorn cupping contain exosomes. OHCT might exert therapeutic effects in RA through multiple signaling pathways and multiple protein targets.

## Introduction

Rheumatoid arthritis (RA) is a systemic autoimmune disease characterized by multiple systemic injuries. It is an inflammatory disorder that primarily involves erosive synovitis and multiple joint destructions (mainly the small joints of hands and feet), causing severe medical and economic burdens [1]. Statistically, RA impacts approximately 0.5–1% of the adult population and presents two- to three-fold more frequently in women than men [2].

Exosomes are cell-secreted nanocarriers (from various cells in body fluids) bearing functional bio-molecules, which act as intracellular communicators, mediating and participating in physiological and pathological processes. With the rise of exosome research, a growing number of studies have recently found that exosomes are closely associated with autoimmune diseases and inflammatory reactions. Through intercellular communication, exosomes regulate inflammation and immune responses in RA [3]. Mature dendritic cells produce exosomes rich in functional macromolecules (such as major histocompatibility complex and co-stimulatory molecules), which effectively stimulate inflammatory reactions in RA by directly activating T cells to induce antigen-specific immune responses [4]. Exosomes play critical roles in immune cells and related tissues through their multiple disease markers. Inhibitor of DNA-binding 1 (Id1) has been found in synovial fluid and exosomes secreted by fibroblast-like synoviocytes (FLS) in RA patients. The Id1 can accelerate the formation of new blood vessels in the inflammatory micro-environment by activating the intracellular c-Jun N terminal kinases signaling pathway, promoting FLS proliferation, and inhibiting IL-6 and IL-8 secretion in immune cells, consequently mediating RA pathogenesis [5]. Morgan et al. isolated CD13-carrying exosomes in RA patients’ plasma, synovial fluid, FLS supernatant, and CD13 were subsequently verified to substantially boost the proliferation and migratory ability of intra-articular FLS, ultimately leading to the FLS pathological proliferation [6].

Furthermore, CD13 can recruit T cells into inflammatory tissue, and T cells subsequently produce various inflammatory factors that promote FLS proliferation, deteriorating the condition of RA patients [7]. The membrane-type TNF (tumor necrosis factor)-α carried by FLS-derived exosomes has been demonstrated to activate PK (protein kinase) B/AKT and NF (nuclear factor)-κB signaling pathways, promote FLS to secrete matrix metalloproteinase 1 (MMP-1) that destroys extracellular matrix and cartilage matrix, and block the intracellular apoptosis pathway to inhibit normal T cells apoptosis [8]. Upon stimulation by T cell-derived exosomes and monocytes in RA patients, FLS can secrete proteins, such as MMP-1, MMP-3, IL (interleukin)-6, IL-8, MCP (monocyte chemoattractant protein)-1, and MCP-2 [9]. Additionally, the TNF-α carried by FLS-derived exosomes can stimulate the NF-κB signaling pathway, enhance the T cell anti-apoptosis ability, and promote T cell-mediated inflammatory response [10].

The OHCT is a widely applied external treatment in Mongolian medicine. The OHCT has been implied to exert a significant therapeutic effect on arthritis and rheumatic immune diseases [11]. But its mechanism of action is still unclear. Therefore, the RA-related exosomal proteins extracted from the body fluids of RA patients by oxhorn cupping can be analyzed and screened for their composition, and preliminary investigations can be conducted for the treatment of the disease by humoral modulation.

## Materials and methods

### Subjects

At the outpatient and inpatient clinics of Inner Mongolia Xilinguole Meng Mongolian General Hospital, 125 RA patients (20–75 ages) recruited between December 2020 and May 2023 received a diagnosis and treatment. The body fluid samples produced by the oxhorn cupping were then taken from 30 individuals who were chosen at random. All subjects signed a written informed consent form and fulfilled the diagnostic, inclusive, and exclusive criteria. This study was approved by the Ethics Committee of Inner Mongolia Xilinguole Meng Mongolian General Hospital (Ethics Nr. 20201201).

### Methods

#### Oxhorn cupping therapy

Based on the physical condition of the subjects, the initial cupping time at the relevant pain area and the lesion site lasted 50 min. Blisters of varying sizes, mainly characterized by light yellow or colorless transparent liquid, were expected to form in the cupping area. Subsequently, the cupping area was thoroughly disinfected using medical iodine and covered with gauze. The following day, cupping was performed in the same area and lasted 40–60 min considering the subjects’ physical condition. The process was subsequently repeated each day until the fluid in the targeted area could no longer be extracted. The treatment was thereafter discontinued. In a short time, the subjects’ condition gradually improved following the wound scabbing and falling off. Based on individual conditions, the process lasted 4–10 days, and the cupping was conducted at 1–5 relevant acupoints. After the wound healed, medical staff initiated cupping in other affected areas considering the patients’ condition. A total of three treatment courses (one per week) were administered.

#### Body fluid collection and exosome isolation

Liquid (body fluid) accumulated in the cupping vessels placed on certain points was collected from RA patients three times during OHCT. The samples were first centrifuged at 2000 g, 4°C, for 30 min. The supernatant was subsequently transferred to a new centrifuge tube, and centrifuged at 10000 g, 4°C, for 45 min. The filtrate was collected by filtering the supernatant through a 0.45 μm filter membrane. The filtrate was then centrifuged for 70 min at 100000 g, 4°C. We removed the supernatant and then resuspended the pellet in 10 mL of pre-cooled 1×PBS, followed by 70 min of centrifugation at 100000 g, 4°C. After removing the supernatant, we resuspended the exosomes in 150 μL pre-cooled 1×PBS. We used 10 μL of exosomes for transmission electron microscope (TEM) observation, 10μL for particle size analysis, and 30 μL for flow cytometry and froze the remaining exosomes at -80°C.

#### Exosome characterization

1. Characterization by transmission electron microscopy (TEM): We removed 10 μL of exosomes, pipette 10 μL of the sample, and added it dropwise onto a copper grid to precipitate for 1 minute, then absorbed the floating liquid on filter paper. Dioxyuranium acetate 10 μL drop was added to the copper mesh to precipitate for 1 min, and the floating liquid was sucked off by filter paper. The sample was dried at room temperature for a few minutes, and then subjected to electron microscopy at 100 kv to obtain the transmission electron microscopy imaging results.
2. Detection of particle size, concentration, and surface potential by nanoparticle tracking analyzer: After diluting the aforementioned exosome suspension 1000 times with ultrapure water, we used a ParticleMetrix nanoparticle tracking analyzer to assess the exosomes concentration (vesicle number/mL), particle size distribution range, and exosomes membrane zeta potential. Exosome concentration was determined as described previously [12].
3. Flow cytometry fluorescent labeling and detection of exosomes: First diluted 30 μL of exosomes into 90 μL. We then transferred 30 μL of the diluted solution into a fresh tube and added 20 μL fluorescent-labeled antibodies (FITC Mouse Anti-Human CD9, FITC Mouse Anti-Human CD63, and FITC Mouse Anti-Human CD81). The samples were subsequently incubated for 30 min in the dark at 37°C after thoroughly mixing. After adding 1 mL of pre-cooled PBS, the samples were centrifuged for 70 min at 4°C, 110000g. The supernatant was carefully removed, and 1 mL of pre-cooled PBS was added. The samples were then centrifuged again for 70 min at 4°C, 110000 x g. We carefully removed the supernatant and resuspended the pellet in 50 μL of pre-cooled 1×PBS. When the sample was measured, the protein index results detected by the NanoFCM instrument could be obtained [13].

#### Label-free quantitative proteomics analysis

During OHCT, fluid samples were obtained from 9 RA patients, and proteomics analysis was conducted at three stages: pret-treatment/post-treatment, pre-treatment/under-treatment, and under-treatment/post-treatment. Each stages had 3 biological replicates, resulting in a total of 9 samples for concentration testing. During sample processing, acetone precipitation was first performed, followed by protein resolubilization, reduction, alkylation, protease digestion, SDS removal, and peptide desalting. The processed peptide samples were then subjected to nanoscale liquid chromatography coupled to tandem mass spectrometry (nano LC-MS/MS).

The liquid chromatography system utilized an enrichment column (3 cm×75 μm) packed with Magic AQ C18 material (particle size = 5 μm, pore diameter = 100Å), as well as an analytical column (10 cm × 75 μm) also packed with Magic AQ C18 material (particle size = 5 μm, pore diameter = 100Å), operated at 800 psi using a pressure injection pool. The electrospray ionization source was equipped with an 8 μm emission tip (New Objective, Woburn, MA) and maintained an ion spray voltage of 2000 V. Samples were loaded onto the enrichment column containing 0.1% formic acid and 5% acetonitrile for 15 minutes. The peptide samples were then separated using a linear gradient (7–35% solvent B, where solvent B is 90% acetonitrile with 0.1% formic acid) at a constant flow rate of 350 nL/min over 60 minutes. Data was acquired using Xcalibur 4.1 software. Full scan data was obtained using an Orbitrap mass analyzer at a resolution of 60000 at 400 m/z, with MS spectra recorded in the 350 to 1800 m/z range. MS/MS data was collected using an Orbitrap mass analyzer at a resolution of 15000 at 400 m/z. The 20 most abundant ions were selected and fragmented by dissociation with 39% normalized collision energy. Singly charged ions and unassigned precursor ions were excluded. During data collection, a dynamic exclusion operation with a 60-second exclusion time was initiated. The lock mass option, enabled by polydimethylsiloxane (m/z 415.12) ions, was used for real-time calibration.

#### Bioinformatics analysis

Proteins with significant differences in abundance identified in proteomic analysis were further examined using the bioinformatics tool. The Proteome Discoverer (PD) (version 2.4.0.305, Thermo Fisher Scientific) and the built-in Sequence HT search engine were used to analyze the original data files. The GO (gene ontology) annotations classify proteins based on biological processes, cellular components, and molecular functions. For each category, differentially expressed proteins (DEPs) enrichment on all identified proteins was tested using a double-tailed Fisher’s exact test. The revised screening criteria for DEPs: Herein, DEPs were screened by statistical approaches based on the Label-Free technology route. Student’s t-test or chi-square test with p<0.05 and fold change ≤0.25 or ≥1.5 were the DEPs screening criteria. Through frequency analysis, the p-value in the chi-square test was evaluated, and the statistical significance level was set at p<0.05. COG (cluster of orthologous groups) databases were used to classify the identified proteins. Additionally, through a two-tailed Fisher’s exact test, the Encyclopedia of Genes and Genomes (KEGG) database was used to identify enrichment pathways, testing the enrichment of all identified proteins by DEPs. Pathways with adjusted p-value <0.05 were deemed statistically significant. According to the KEGG website, these pathways were classified into hierarchical categories. The protein-protein interactions (PPI) were analyzed via the STRING database and Cytoscape 3.9.0 software.

#### Detection of DEPs in serum

EDTA anticoagulant tubes (1 tube in total) were used to collect 2 mL of blood. The blood sample was centrifuged for 10 min at 2000 rpm after being kept at room temperature for 0.5 h. The supernatant was then transferred to a 1.5 mL labeled EP tube and stored at -80°C for testing. The cyclin-dependent kinase 1 (CDK1), CD44, integrn beta-5 (ITGB5), and integrin alpha-5 (ITGA5) levels in all samples were determined using enzyme-linked immunosorbent assay (ELISA) kits (Jiangsu Zeyu Biotechnology Co., Ltd).

#### Statistical analysis

The data obtained were transferred to the Excel window (version 5.2.1, WPS) and imported to the Prism9 software for statistical analysis and icon design. One-way ANOVA was performed for inter-group comparisons, and the Receiver Operating Characteristic analysis was conducted to test the overall diagnostic accuracy. Results with p<0.05 were considered statistically significant.

## Results

### Morphological characteristics of exosomes

**Fig 1:**
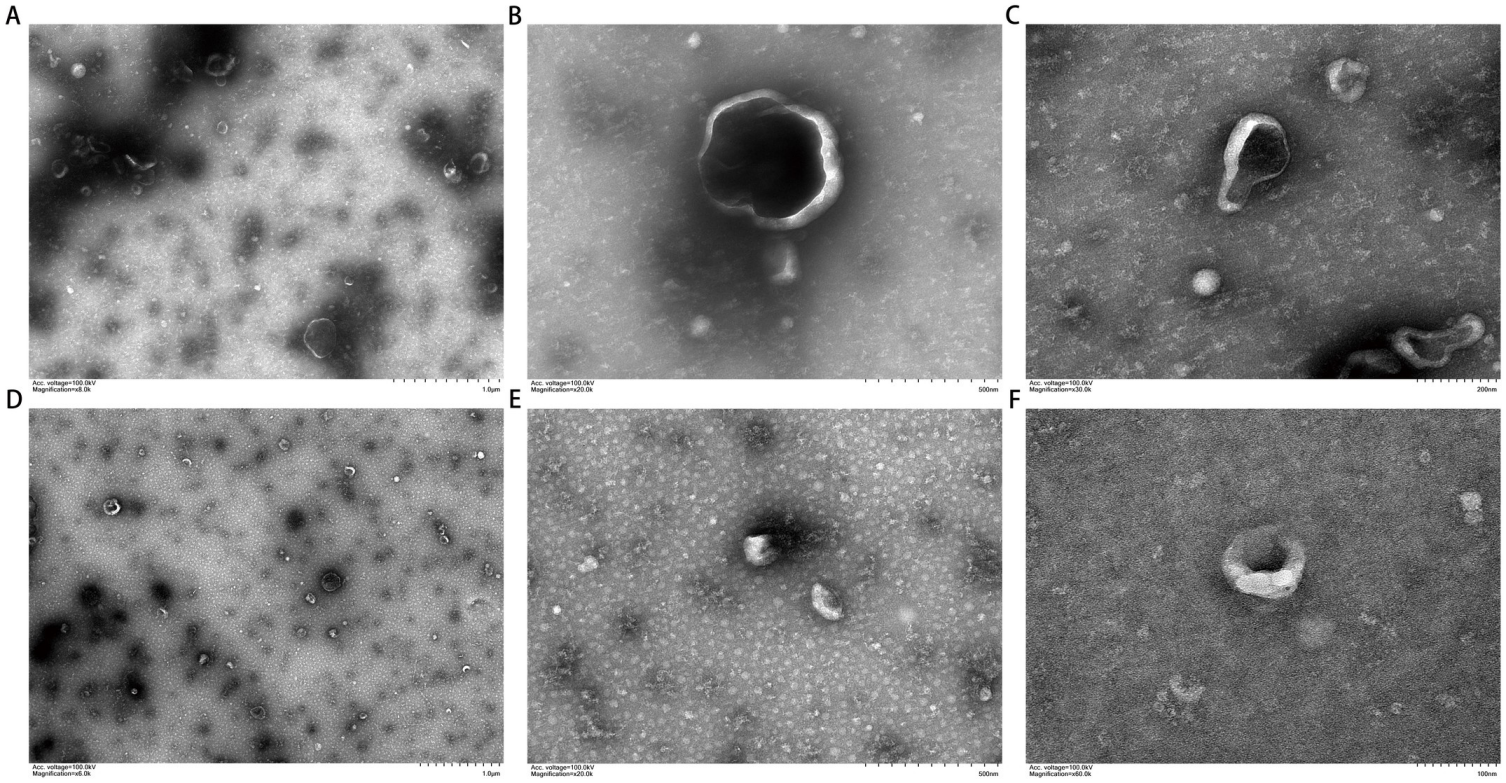
TEM of exosomes in body fluid samples obtained by oxhorn cupping. (A) 1.0 μm before treatment; (B) 500 nm before treatment; (C) 200 nm before treatment; (D) 1.0 μm after treatment; (E) 500nm after treatment; (F) 200 nm after treatment.

**Fig 2:**
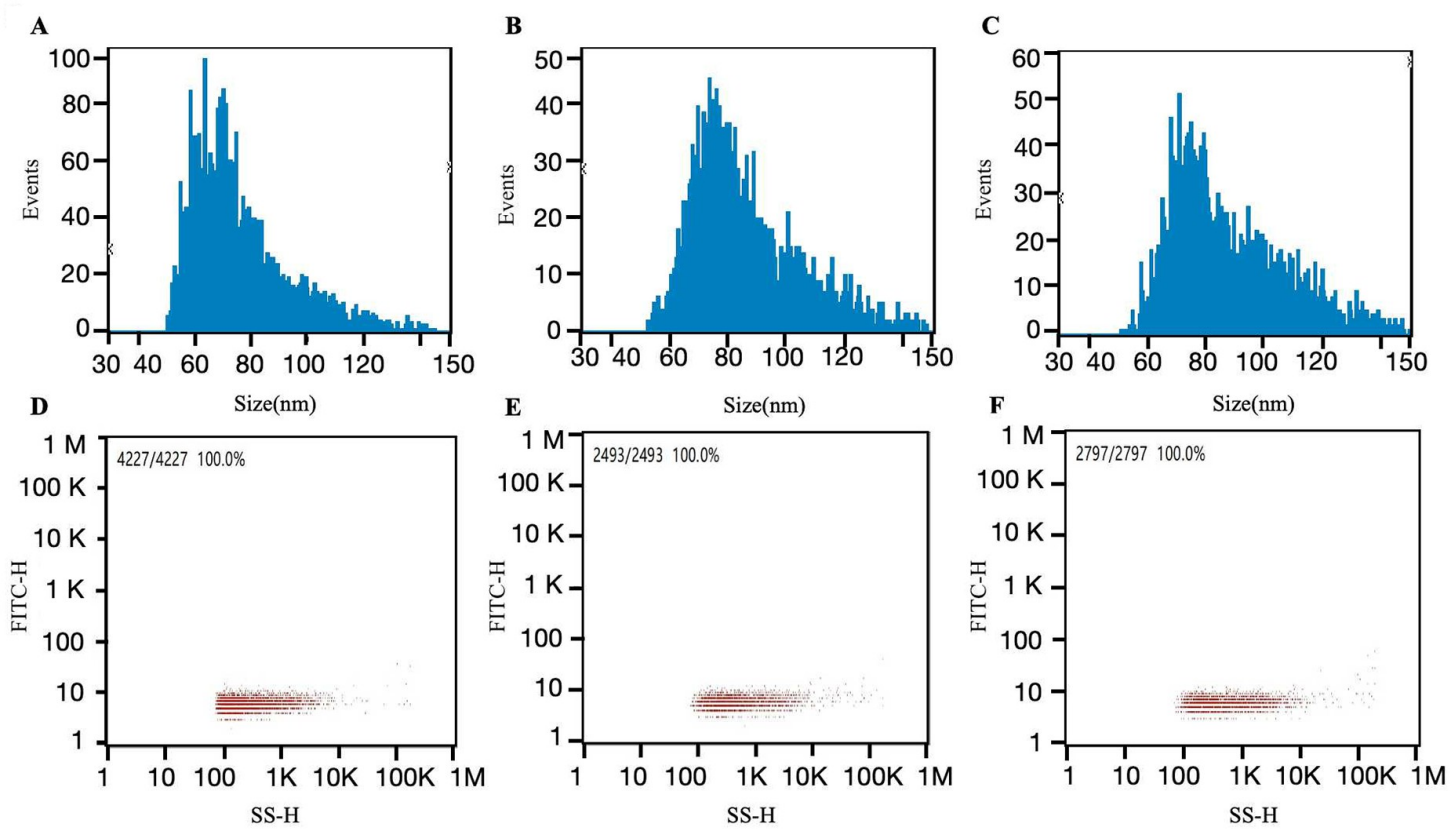
Exosome size distributions in body fluid samples obtained by oxhorn cupping. (A) Average exosome size before treatment (nm): 76.13; (B) Average exosome size during treatment (nm): 85.65; (C) Average exosome size after treatment (nm): 87.63; (D) Exosome concentration before treatment (/mL): 5.27E+10; (E) Exosome concentration during treatment (/mL): 2.58E+10; (F) Exosome concentration after treatment (/mL): 4.34E+10.

The TEM images revealed an excellent dispersion of the extracted exosomes, most of which were cup-shaped circular or quasi-circular membrane vesicles. The vesicles exhibited a dual membrane structure with low electron density components in the center, a relatively concentrated distribution, and clear boundaries (Fig 1). The average exosome size was 76.13 nm (5.27E+10 /mL (Fig 2).

### Fluorescent labeling and flow cytometry results of exosome samples

**Fig 3:**
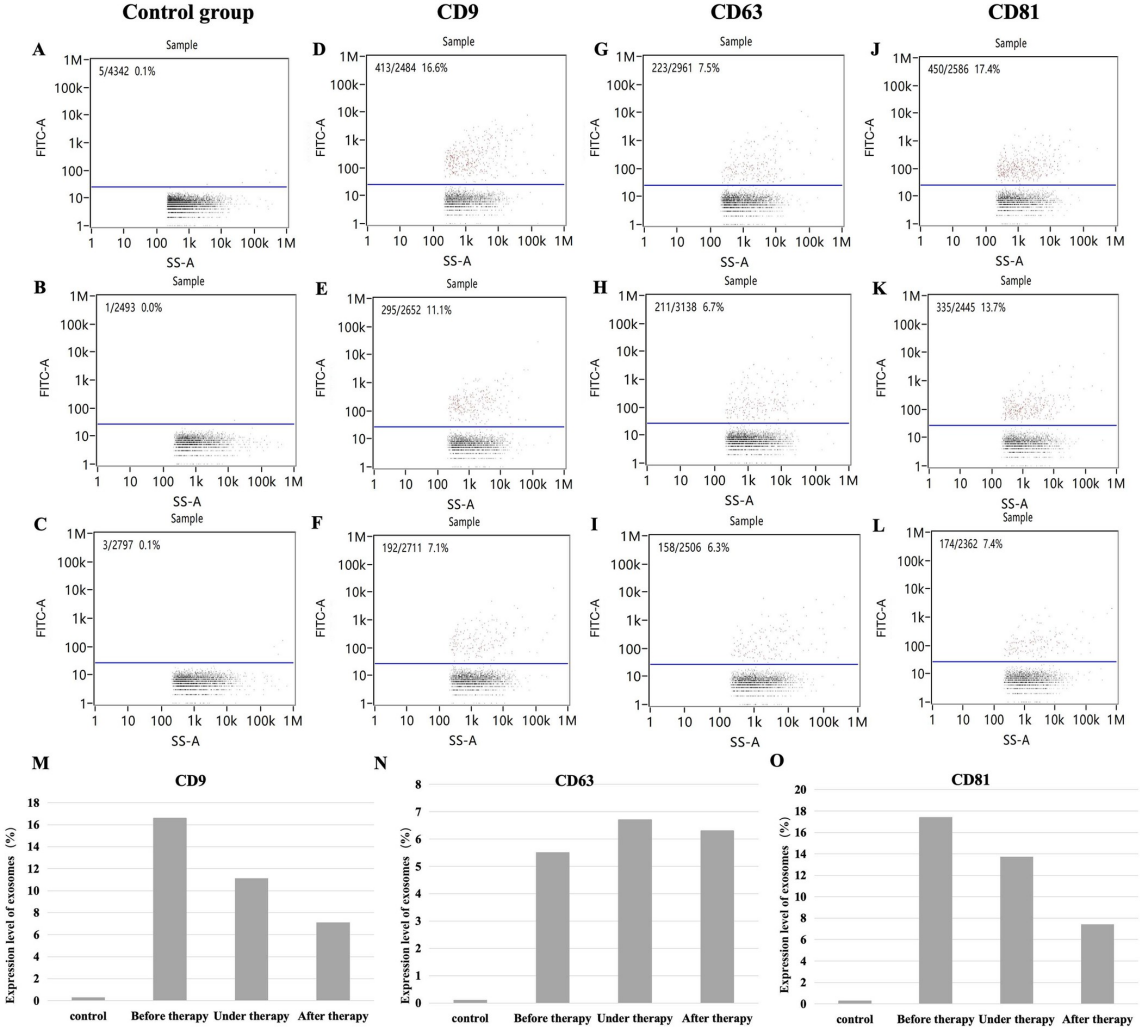
Flow cytometry detection of surface protein expression on body fluid exosomes obtained by oxhorn cupping. (A) Intra-group isotype control FITC-IgG (0.1%) before treatment; (B) Intra-group isotype control FITC-IgG (0.0%) during treatment; (C) Intra-group isotype control FITC-IgG (0.1%) after treatment; (D) CD9 (16.6%) before treatment; (E) CD9 (11.1%) during treatment; (F) CD9 (7.1%) after treatment; (G) CD63 (7.5%) before treatment; (H) CD63 (6.7%) during treatment; (I) CD63 (6.3%) after treatment; (J) CD81 (17.4%) before treatment; (K) CD81 (13.7%) during treatment; (L) CD81 (7.4%) after treatment; (M) Surface CD9 content before treatment and after treatment; (N) Surface CD63 content before treatment and after treatment; (O) Surface CD81 content before treatment and after treatment.

With FITC-IgG as the isotype control (0.1% pre-treatment) the positive rate of surface protein expression of exosomes in body fluid was as follows: CD9 (16.6%), CD63 (7.5%), and CD8 (17.4%). Additionally, with FITC-IgG as the isotype control (0.0% under-treatment), the positive rate of surface protein expression of exosomes in body fluid was as follows: CD9 (11.1%), CD63 (6.7%), and CD8 (13.7%). Finally, with FITC-IgG as the isotype control (0.1% post-treatment), the positive rate of surface protein expression of exosomes in body fluid was as follows: CD9 (7.1%), CD63 (6.3%), and CD8 (7.4%) (Fig 3).

### Protein quantification and differential analysis

**Fig 4:**
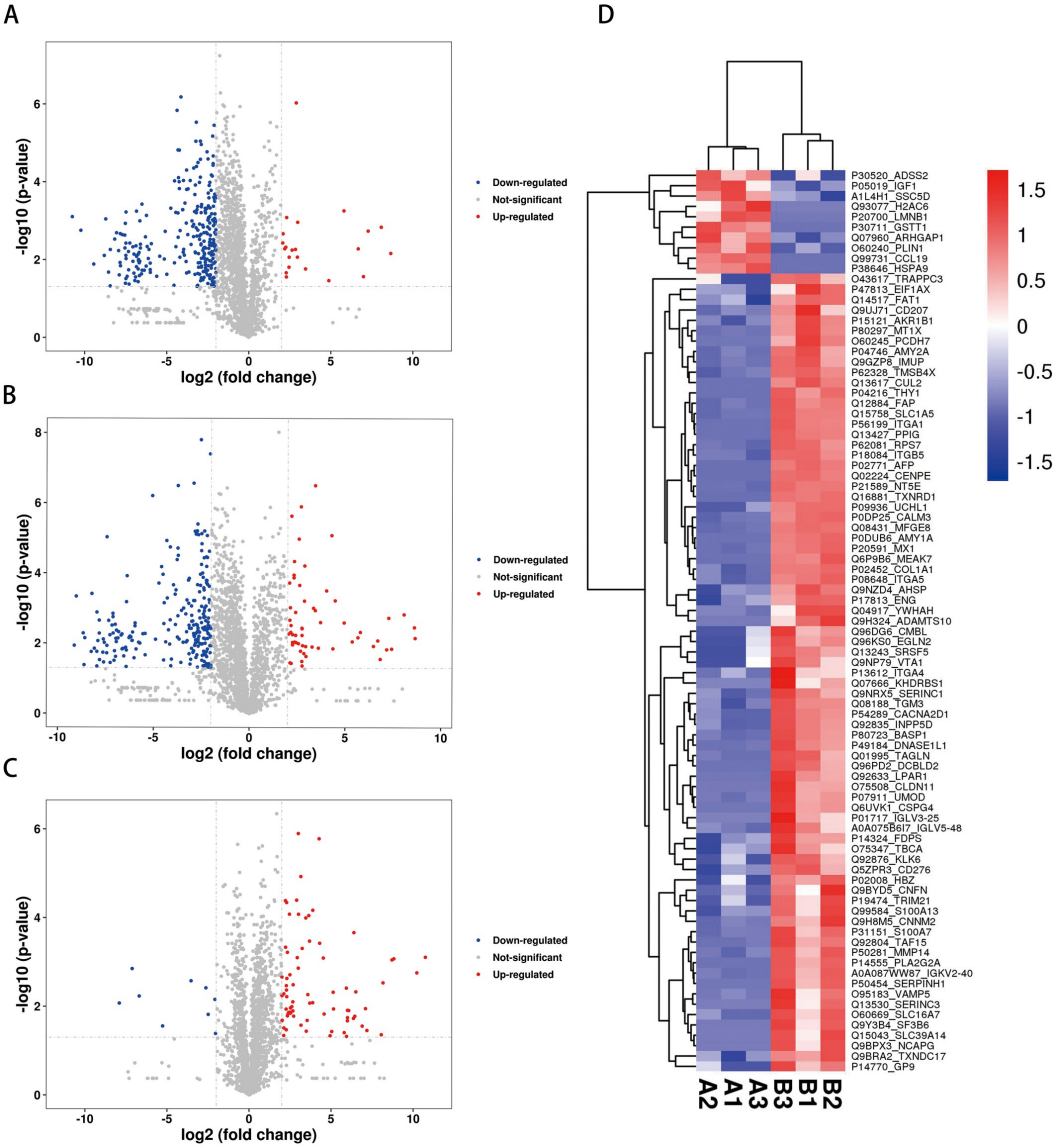
Identification of differentially expressed proteins. (A-C) Volcano plot of the DEPs. (A) under-treatment/post-treatment; (B) pre-treatment/post-treatment; (C) pre-treatment/under-treatment. In volcano plots red represents significantly upregulated DEPs, blue represents significantly downregulated DEPs, and gray represents non-significantly DEPs; (D) Hierarchical clustering heatmap showing the DEPs between pre-treatment and under-treatment. The horizontal axis represents various groups, and the vertical axis represents the DEPs compared to that group. The color blocks at different positions show the relative expression levels of the corresponding proteins, with red showing high expression and blue showing low expression.

The protein samples were analyzed using a label-free quantification method, and nano-LC/MS was used to study and compare the DEPs in body fluid exosomes of RA patients before, during, and after OHCT. In our study, we found 2197 proteins and 14423 peptides. Furthermore, we quantified 8077 DEPs. Between the pre-treatment and post-treatment stages, we identified 300 DEPs (58 up-regulated and 242 down-regulated). Between the pre-treatment and under-treatment stages, we found 87 DEPs (77 up-regulated and 10 down-regulated). While, between the under-treatment and post-treatment stages, we identified 303 DEPs (22 up-regulated and 281 down-regulated). The label-free proteomics analysis showed a p-value < 0.05 and a fold change < 0.67 in the Student’s t-test. Volcano graphs (Fig 4A–4C) and the heatmap (Fig 4D) showed DEPs between the treatment stages.

### Functional analysis and subcellular localization of differentially expressed proteins

**Fig 5:**
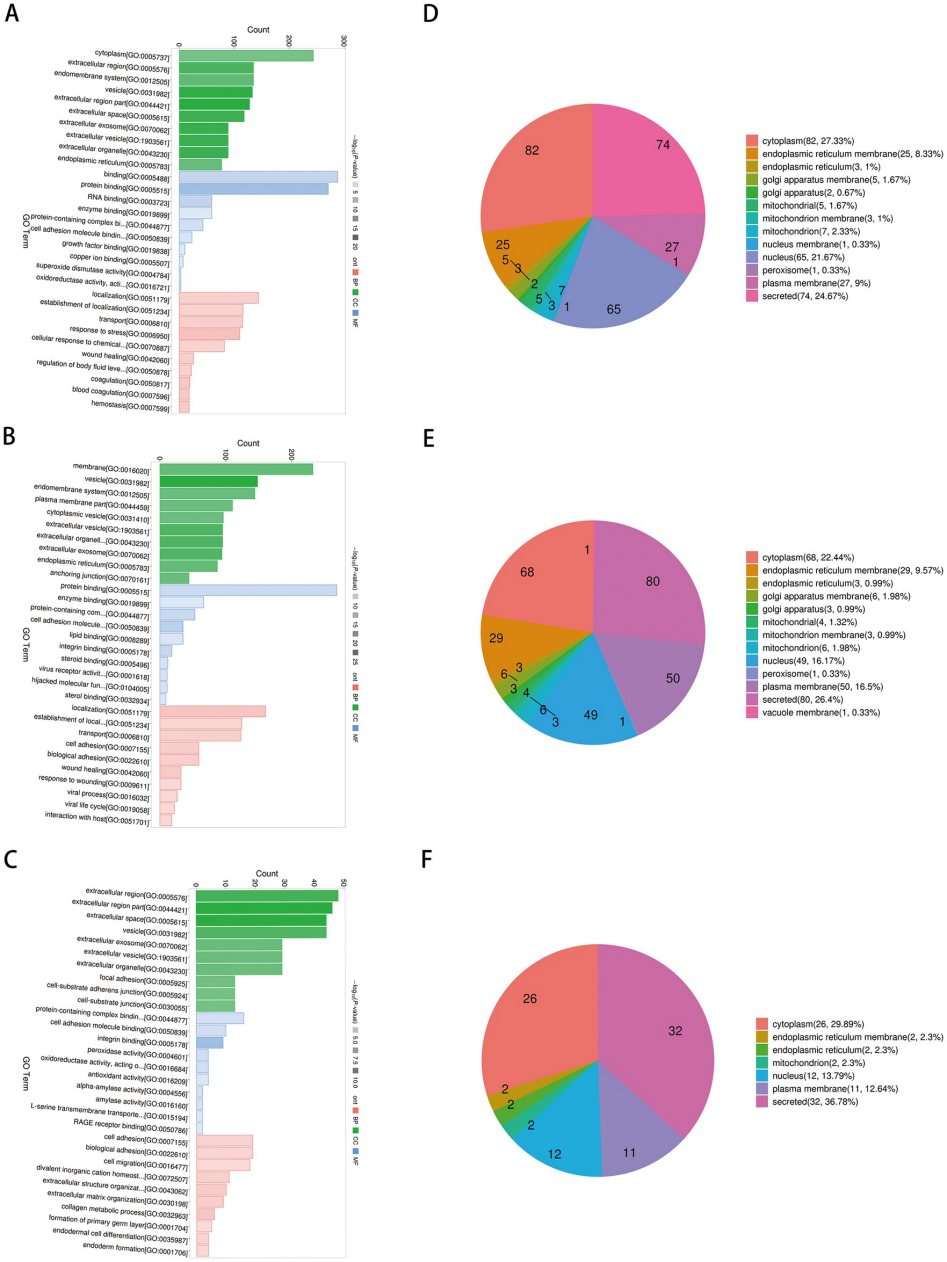
GO annotation and subcellular localization analysis. (A-C) GO annotation classification analysis in biological process (BP), cellular component (CC), and molecular function (MP). (A) pre-treatment/post-treatment, (B) under-treatment/post-treatment, and (C) pre-treatment/under-treatment. The horizontal and vertical axes represent the GO term and the number of DEPs mapped, respectively. Red represents BP annotation information, green represents CC annotation information, and blue represents MF annotation information.; (D-F) Classification of DEPs based on subcellular localization. (D) pre-treatment/post-treatment, (E) under-treatment/post-treatment; (F) pre-tretreatment/under-treatment.

The DEPs determined across the three stages (pre-treatment/post-treatment, pre-treatment/under-treatment, and under-treatment/post-treatment) were analyzed for GO functional enrichment analysis. In biological processes, the DEPs were mainly enriched in response to stress and focal adhesion. In cellular components, the main focus is on extracellular region, exosome, and vesicle. In molecular function, DEPs were involved in protein binding and protein containing complex binding (Fig 5A–5C). The DEPs were located mainly in the cytoplasm, plasma membrane, endoplasmic reticulum membrane, and secreted (Fig 5D–5F).

**Fig 6:**
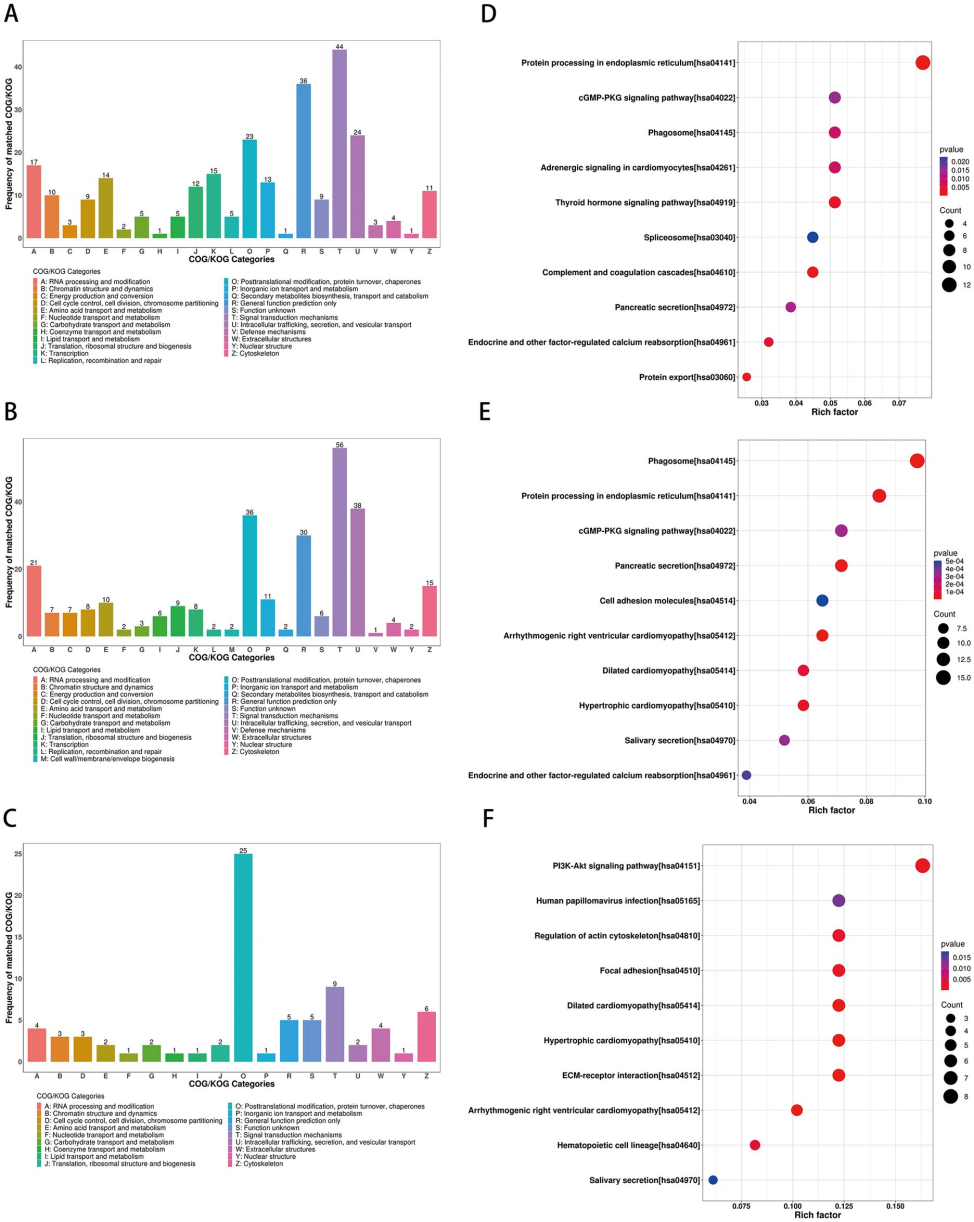
COG/KOG classification and KEGG pathway analysis. (A-C) COG/KOG functional classification of the DEPs analysis. (A) pre-treatment/post-treatment; (B) under-treatment/post-treatment; (C) pre-treatment/under-treatment. Herein, the X and Y axes in COG analysis results refer to the classification of COG and the frequency number of COG/KOG, respectively; (D-F) KEGG enrichment analysis of the DEPs. (D) pre-treatment/post-treatment, (E) under-treatment/post-treatment, (F) pre-treatment/under-treatment. The size of the circle represents the number of DEPs in the mapping pathway, and the larger circle represents more DEPs. The color of the circle represents the p-value. As the color deepens, the p-value decreases.

According to the results of COG/KOG functional classification, the DEPs were mainly related to intracellular trafficking, secretion, vesicular transport, posttranslational modification, protein turnover, and chaperones, as well as signal transduction mechanisms (Fig 6A–6C).

KEGG pathway enrichment bubble chart analyses revealed the DEPs were enriched in PI3K-Akt signaling pathway, focal adhesion, protein processing in endoplasmic reticulum, phagosome, complement, and coagulation cascade (Fig 6D–6F).

### Protein-protein interaction network analysis

**Fig 7:**
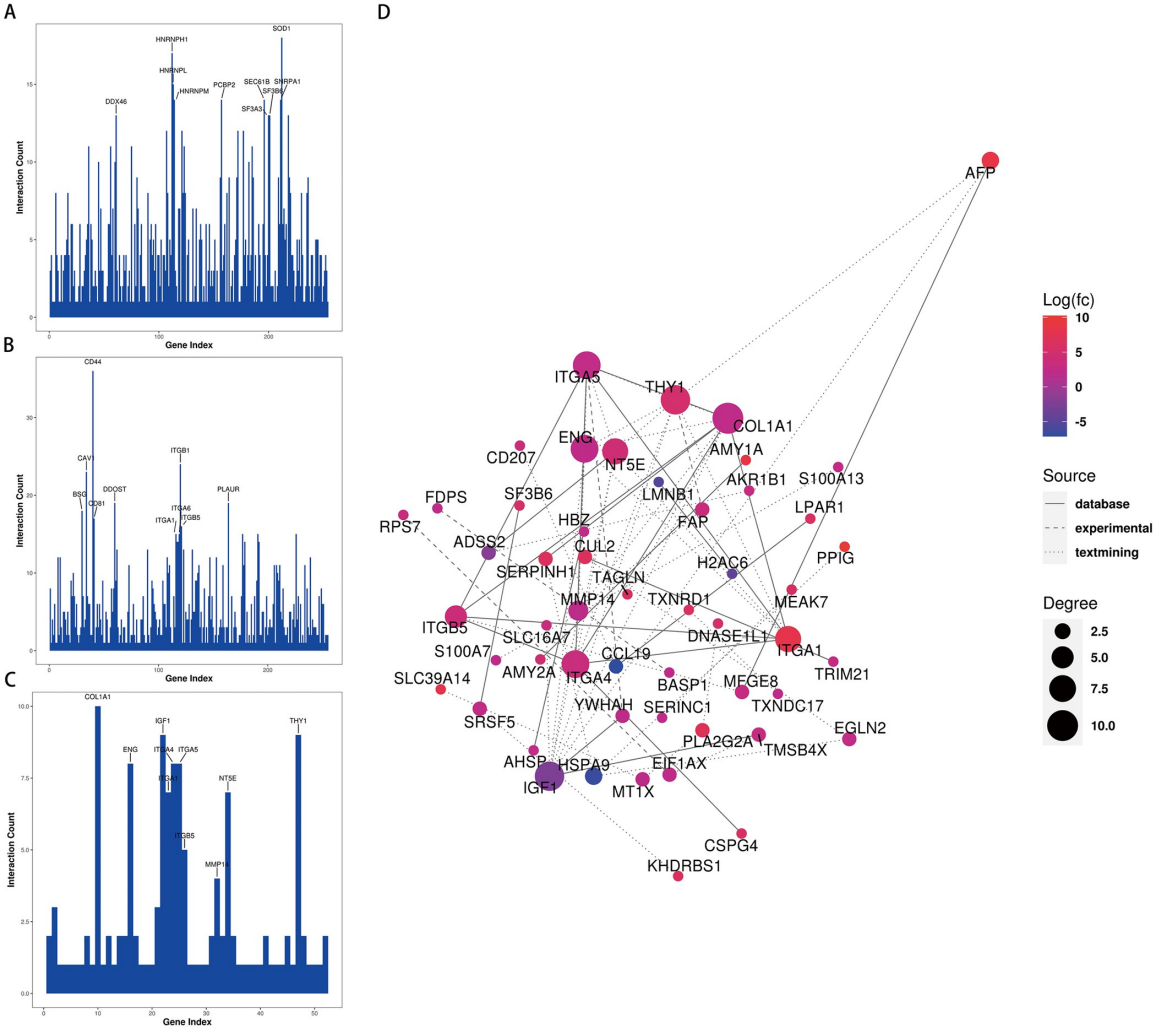
PPI network analysis. (A-C) Protein interaction connectivity histogram. (A) pre-treatment/post-treatment, (B) under-treatment/post-treatment, (C) pre-treatment/under-treatment; (D) Diagram of PPI networks showing the DEPs between pre-treatment and under-treatment. The color in the figure represents the expression level of DEPs, red represents a significant up-regulation, and blue represents a significant down-regulation; the size of the circle represents the connectivity of the differentially expressed proteins, the higher the connectivity, the larger the circle; The type of connection represents the source of the interaction relationship. The solid line represents the interaction relationship from the database, the dotted line represents the interaction relationship from the experiment, and the dotted line represents the interaction relationship from text mining.

The STRING online database (with a confidence score > 0.4 and a degree > 5 as cut-off criteria) was used to determine the relationships among the DEGs. The PPI network was subsequently constructed using Cytoscape 3.9.0 software to analyze and visualize the significance of the target proteins. The analysis revealed that ITGA5, ITGA4, ENG, MMP14, SERPINH1, THY1, TAGLN, ITGA1, IGF1, and ITGB5 were centrally located in the network module and showed high connectivity with other proteins (Fig 7).

### Detection of DEPs in serum by ELISA

**Fig 8:**
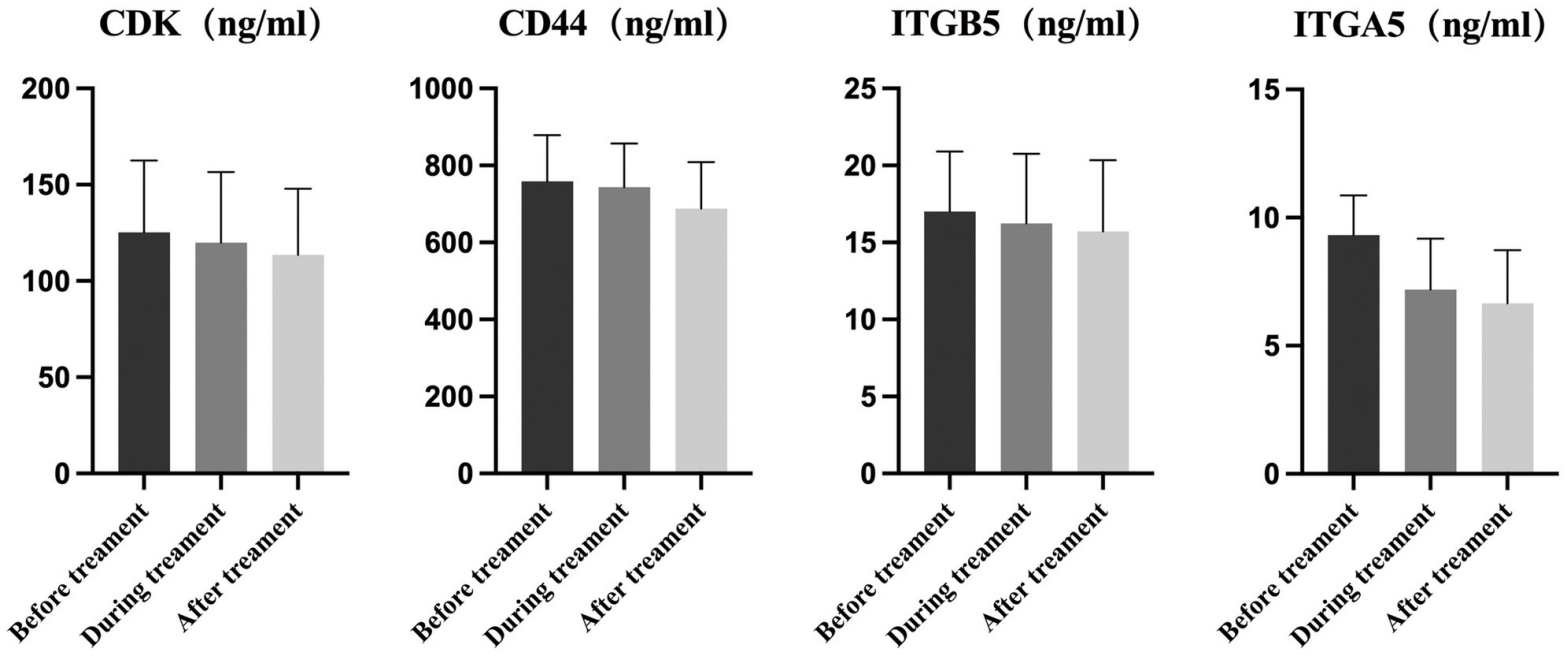
ELISA of the key proteins.

The protein expression levels of CDK1, ITGA5, ITGB5, and CD44 levels in serum were detected by ELISA to verify the results of proteomics analysis. Although a slight decrease was observed in the levels of these proteins during and after treatment compared to those before treatment, no statistically significant differences were found (Fig 8).

## Discussion

As a low-cost therapeutic technique, cupping therapy is one of the most common complementary therapies, and its clinical efficacy is remarkable, but its mechanism of action is still unclear. Cupping therapy is suitable for many diseases, especially for joint conditions. Researchers proposed that the main action of cupping therapy is to enhance the blood circulation and to remove toxins and waste from the body [14].

Cups made of various materials have been used in Asian countries since ancient times. As written in historical and medical sources, Mongolians have been used cups made from oxhorns for hundreds of years. Horn cupping creates strong negative pressure on the body surface to form blisters and pull out body fluid [11].

Exosomes are nanosized (40–100 nm) extracellular particles found in mammals. They are a transporter of lipids, proteins, and genetic material involved in mammalian cell signaling and biological process. Exosomes are found in body fluids such as plasma, saliva, urine, cerebrospinal fluid, ascites, and breast milk. Studies demonstrated the pivotal role of exosomes in the diagnosis and treatment of various diseases such as tumor, metabolic disorders, cardiovascular diseases, neurological conditions, and kidney diseases [15]. Moreover, exosomes have been shown to play an important role in RA-related joint inflammation [16].

In the present study, we isolated and characterized exosomes from the liquid (body fluid) extracted in the cupping vessels during OHCT. We found that exosomes isolated from the body fluids of patients showed the expected size (76.13 nm) and expressed the marker proteins CD63, CD81, and CD9.

Further, we identified 300 DEPs (58 up-regulated and 242 down-regulated) between the pre-treatment and post-treatment stages in body fluid exosomes using label-free quantitative proteomics analysis. GO enrichment analysis revealed t**hat** the DEPs were highly related to protein binding, focal adhesion, extracellular region, exosome, and vesicles.

KEGG pathway enrichment showed that the DEPS are mainly related to PI3K-Akt signaling pathway, focal adhesion, protein processing in endoplasmic reticulum, phagosome, complement, and coagulation cascade.

Studies have proven that cell adhesion plays an essential role in the generation of chronic synovitis during RA [17]. Interestingly, PI3K-Akt signaling pathway is involved in the focal adhesion [18]. It has been proven that the PI3K/AKT signaling pathway is correlated with the occurrence and development of RA. It can participate in the unusual proliferation of FLS cells and synovial inflammation by stimulating the expression of inflammatory cytokines such as IL-1β, IL-6, IL-17, IL-21, IL-22, and TNF-α, which constitute the most important pathogenesis of RA pathological changes [19]. We speculate that OHCT can alleviate RA by regulating multiple pathways, including focal adhesion and the PI3K-Akt signaling pathway.

In addition, according to PPI network analysis, ITGA1, ITGA4, ITGA5, ITGB5, ENG, MMP14, SERPINH1, THY1, TAGLN, and IGF1 located in the center of the network module and highly connected to other proteins. Furthermore, ELISA results showed that a slight decrease in the selected proteins such as CDK1, ITGA5, ITGB5, and CD44 during and after treatment compared to those before treatment.

ITGA5, ITGA4, ITGA1, and ITGB5 are the member of integrin family of transmembrane receptors. Integrins play an important role in cell adhesion to the extracellular matrix and other cells. Integrins activate focal adhesion kinase (FAK) and Src-family kinases, and subsequently stimulate downstream signaling cascades such as PI3K-Akt signaling. Furthermore, increased integrin ligation enhances growth factor and cytokine signaling and induces the expression of MMP, which further degrade extracellular matrix molecules [20].

MMP synthesized by chondrocytes and synovial fibroblasts are considered the main enzymes responsible for cartilage destruction. Studies showed that targeting MMP14 is important for treatment of RA [21].

Endoglin encoded by ENG gene is a transmembrane accessory receptor for TGFβ. Endoglin may control cell adhesion and migration by regulating the composition of focal adhesion complexes and can regulate angiogenesis [22].

IGF1, a member of insulin-like growth factor family of ligands is believed to play an important role in the maintenance of the steady-state metabolism of cartilage. Studies reported high IGF1 levels in both plasma and synovial fluid of RA patients compared to controls [23].

HSP47, a SERPINH1 gene encoded protein was reported as RA-related antigen protein from a human chondrosarcoma-derived chondrocytic cell line. The altered localization of HSP47 to the cell surface or the secretion into the blood may be used as the marker of RA [24].

THY1 is a cell surface protein and expressed on the surfaces of fibroblasts, and participates in cell growth and differentiation. THY1-positive fibroblasts are aggregated in the synovia of patients with RA [25].

TAGLN (transgelin) is a TGFβ-inducible gene that functions as an actin-crosslinking protein of the calponin family. Transgelin 2 expression has been shown to greatly changed in patients with RA treated with infliximab [26].

CDK1 (cyclin-dependent kinase) is one of 20 kinases of CDK protein family and is the only CDK in mammals is essential for cell cycle progression. Recent studies reported that cell cycle regulation can be a new therapeutic approach for treatment of RA as overgrowth of synovial fibroblasts to form a hyperplastic pannus. This anti-proliferative treatment focuses on CDK1 as a potential target [27].

CD44 is a transmembrane glycoprotein belonging to the family of adhesion molecules. Expression of CD44 and its variants in the synovial tissues of patients with RA is associated with cartilage damage. Activation of CD44 triggers the activation of many downstream pathways such as mitogen activated protein kinase and PI3K-Akt in order to mediate cell migration and proliferation. Targeting CD44 has emerged as a promising therapeutic strategy [28].

Taken together these key DEPs are closely associated with pathophysiology of RA and OHCT regulates RA through multiple targets and multiple pathways.

## Conclusion

In this study, we determined that OHCT might regulate multiple targets including ITGA1, ITGA4, ITGA5, ITGB5, ENG, MMP14, SERPINH1, THY1, TAGLN, IGF1, CDK1 and CD44, etc., focal adhesion and the PI3K-Akt pathway and other pathways to alleviate RA.

### Clinical trial registration information

1. Registration name: Effect of traditional Mongolian medicine horn cupping therapy (OHCT) on the treatment of rheumatoid arthritis and its proteomics;
2. Number: ChiCTR2300071505;
3. Location: World Health Organization International Clinical Trial Registration Platform Level 1 Registration Agency—China Clinical Trial Registration Center.
4. Retrospectively registered

## References

[1] Guo Q, Wang Y, Xu D, Zhan X, Li Y, Leng X. Rheumatoid arthritis: pathological mechanisms and modern pharmacological therapies. Bone Res. 2018;6:15. 29736302 10.1038/s41413-018-0016-9 PMC5920070

[2] Venetsanopoulou AI, Alamanos Y, Voulgari PV, Drosos AA. Epidemiology and risk factors for rheumatoid arthritis development. Mediterr J Rheumatol. 2023;34(4):404–13. doi: 10.31138/mjr.301223.eaf 38282942 PMC10815538

[3] Zhang B, Zhao M, Lu Q. Extracellular vesicles in rheumatoid arthritis and systemic lupus erythematosus: Functions and applications. Front Immunol. 2021;11:575712. doi: 10.3389/fimmu.2020.575712 33519800 PMC7841259

[4] Sprent J. Direct stimulation of naïve T cells by antigen-presenting cell vesicles. Blood Cells Mol Dis. 2005;35(1):17–20. 15932799 10.1016/j.bcmd.2005.04.004

[5] Ohara RA, Edhayan G, Rasmussen SM, Isozaki T, Remmer HA, Lanigan TM, et al. Citrullinated Inhibitor of DNA Binding 1 is a novel autoantigen in rheumatoid arthritis. Arthritis Rheumatol. 2019;71(8):1241–51. doi: 10.1002/art.40886 30861322 PMC6663620

[6] Du Y, Lu C, Morgan RL, Stinson WA, Campbell PL, Cealey E, et al. Angiogenic and arthritogenic properties of the soluble form of CD13. J Immunol. 2019;203(2):360–9. doi: 10.4049/jimmunol.1801276 31189572 PMC6644048

[7] Morgan R, Endres J, Behbahani-Nejad N, Phillips K, Ruth JH, Friday SC, et al. Expression and function of aminopeptidase N/CD13 produced by fibroblast-like synoviocytes in rheumatoid arthritis: role of CD13 in chemotaxis of cytokine-activated T cells independent of enzymatic activity. Arthritis Rheumatol. 2015;67(1):74–85. doi: 10.1002/art.38878 25219368 PMC4280337

[8] Du F, Lü LJ, Teng JL, Shen N, Ye P, Bao CD. T-614 alters the production of matrix metalloproteinases (MMP-1 and MMP-3) and inhibits the migratory expansion of rheumatoid synovial fibroblasts, in vitro. Int Immunopharmacol. 2012;13(1):54–60. doi: 10.1016/j.intimp.2012.03.003 22446297

[9] Rana AK, Li Y, Dang Q, Yang F. Monocytes in rheumatoid arthritis: Circulating precursors of macrophages and osteoclasts and, their heterogeneity and plasticity role in RA pathogenesis. Int Immunopharmacol. 2018;65:348–59. doi: 10.1016/j.intimp.2018.10.016 30366278

[10] Jang LK, Lee ZH, Kim HH, Hill JM, Kim JD, Kwon BS. A novel leucine-rich repeat protein (LRR-1): potential involvement in 4-1BB-mediated signal transduction. Mol Cells. 2001;12(3):304–12. 11804328

[11] Na R, Su R, Du L, Lan H, Bu R. [Clinical study on the treatment of rheumatoid arthritis with traditional Mongolian medicine]. J Med Pharm Chin Minorities. 2020 July;26(07):34–7. Chinese.

[12] Tian Y, Gong M, Hu Y, Liu H, Zhang W, Zhang M, et al. Quality and efficiency assessment of six extracellular vesicle isolation methods by nano-flow cytometry. J Extracell Vesicles. 2019;9(1):1697028. doi: 10.1080/20013078.2019.1697028 31839906 PMC6896440

[13] Wei H, Qian X, Xie F, Cui D. Isolation of exosomes from serum of patients with lung cancer: a comparison of the ultra-high speed centrifugation and precipitation methods. Ann Transl Med. 2021;9(10):882. doi: 10.21037/atm-21-2075 34164516 PMC8184444

[14] Al-Bedah AMN, Elsubai IS, Qureshi NA, Aboushanab TS, Ali GIM, El-Olemy AT, et al. The medical perspective of cupping therapy: Effects and mechanisms of action. J Tradit Complement Med. 2018;9(2):90–7. doi: 10.1016/j.jtcme.2018.03.003 30963043 PMC6435947

[15] Aheget H, Mazini L, Martin F, Belqat B, Marshal JA, Benabellah K. Exosomes: their role in pathogenesis, diagnosis and treatment of diseases. Cancers (Basel). 2020;13(1):84. doi: 10.3390/cancers13010084 33396739 PMC7795854

[16] Heydari R, Koohi F, Rasouli M, Rezaei K, Abbasgholinejad E, Bekeschus S, et al. Exosomes as rheumatoid arthritis diagnostic biomarkers and therapeutic agents. Vaccines (Basel). 2023;11(3):687. doi: 10.3390/vaccines11030687 36992270 PMC10057381

[17] Liao HX, Haynes BF. Role of adhesion molecules in the pathogenesis of rheumatoid arthritis. Rheum Dis Clin North Am. 1995;21(3):715–40. 8619096

[18] Reif S, Lang A, Lindquist JN, Yata Y, Gabele E, Scanga A, et al. The role of focal adhesion kinase-phosphatidylinositol 3-kinase-akt signaling in hepatic stellate cell proliferation and type 1 collagen expression. J Biol Chem. 2003;278(10):8083–90. 12502711 10.1074/jbc.M212927200

[19] Ding Q, Hu W, Wang R, Yang Q, Zhu M, Li M, et al. Signaling pathways in rheumatoid arthritis: implications of targeted therapy. Signal Transduct Target Ther. 2023;8(1):68. 36797236 10.1038/s41392-023-01331-9 PMC9935929

[20] Lowin T, Straub RH. Integrins and their ligands in rheumatoid arthritis. Arthritis Res Ther. 2011;13(5):244. doi: 10.1186/ar3464 22077951 PMC3308078

[21] Chen Z, Wang H, Xia Y, Yan F, Lu Y. Therapeutic potential of mesenchymal cell-derived miRNA-150-5p-expressing exosomes in rheumatoid arthritis mediated by the modulation of MMP14 and VEGF. J Immunol. 2018;201(8):2472–82. doi: 10.4049/jimmunol.1800304 30224512 PMC6176104

[22] Lebrin F, Goumans M, Jonker L, Carvalho RL, Valdimarsdottir G, Thorikay M, et al. Endoglin promotes endothelial cell proliferation and TGF-β/ALK1 signal transduction. EMBO J. 2004;23(20):4018–28. 15385967 10.1038/sj.emboj.7600386 PMC524335

[23] Skarlis C, Nezos A, Mavragani CP Koutsilieris M. The role of insulin growth factors in autoimmune diseases. Ann Res Hosp. 2019;3:10.

[24] Hattori T, Kubota S, Yutani Y, Fujisawa T, Nakanishi T, Takahashi K, et al. Change in cellular localization of a rheumatoid arthritis-related antigen (RA-A47) with downregulation upon stimulation by inflammatory cytokines in chondrocytes. J Cell Physiol. 2001;186(2):268–81. doi: 10.1002/1097-4652(200002)186:2&lt;168::aid-jcp1022&gt;3.0.co;2-m 11169453

[25] Hu X, Li M, Zhang Y, Sang K, Zhang Y, Li W, et al. An innovative immunotherapeutic strategy for rheumatoid arthritis: Selectively suppressing angiogenesis and osteoclast differentiation by fully human antibody targeting thymocyte antigen-1. Exp Cell Res. 2023;424(1):113490. doi: 10.1016/j.yexcr.2023.113490 36706943

[26] Nozawa K, Fujishiro M, Takasaki Y, Sekigawa I. Inhibition of rheumatoid arthritis by blocking connective tissue growth factor. World J Orthop. 2014;5(5):653–9. doi: 10.5312/wjo.v5.i5.653 25405094 PMC4133473

[27] Sekine C, Sugihara T, Miyake S, Hirai H, Yoshida M, Miyasaka N, et al. Successful treatment of animal models of rheumatoid arthritis with small-molecule cyclin-dependent kinase inhibitors. J Immunol. 2008;180(3):1954–61. doi: 10.4049/jimmunol.180.3.1954 18209094

[28] Quadri MM. Targeting CD44 receptor pathways in degenerative joint diseases: involvement of proteoglycan-4 (PRG4). Pharmaceuticals (Basel). 2023;16(10):1425. doi: 10.3390/ph16101425 37895896 PMC10609794

